# Historical biogeography reveals new independent evolutionary lineages in the *Pantosteus plebeius-nebuliferus* species-group (Actinopterygii: Catostomidae)

**DOI:** 10.1186/s12862-018-1286-y

**Published:** 2018-11-20

**Authors:** Diushi Keri Corona-Santiago, Omar Domínguez-Domínguez, Llanet Tovar-Mora, José Ramón Pardons-Blas, Yvonne Herrerías-Diego, Rodolfo Pérez-Rodríguez, Ignacio Doadrio

**Affiliations:** 1 Departamento de Biodiversidad y Biología Evolutiva, Museo Nacional de Ciencias Naturales, CSIC. c/José Gutiérrez Abascal, 2, 28006 Madrid, Spain; 2 Laboratorio de Biología Acuática, Facultad de Biología, Universidad Michoacana de San Nicolás de Hidalgo, Morelia, Michoacán Mexico; 3 Facultad de Biología, Universidad Michoacana de San Nicolás de Hidalgo, Morelia, Michoacán Mexico

**Keywords:** *cytb*, *GHI*, Evolutionary history, *Pantosteus plebeius-nebuliferus* species-group, Sierra madre occidental, Biogeographic patters, Independent evolutionary history

## Abstract

**Background:**

The *Pantosteus plebeius-nebuliferus* species-group is a group of freshwater fishes distributed in endo- and exorheic drainage basins in the Mexican Sierra Madre Occidental mountain range system and central North Mexico. The geological history of this region is considered an important factor in explaining the evolutionary history of low vagility animals like freshwaters fishes. The aim of this study was to examine the phylogenetic relationships and describe the evolutionary history of the species-group. We hypothesized that the genetic structure and distribution of the main clades of *Pantosteus plebeius-nebuliferus* are associated with the geological history of Northern Mexico. To this end, we obtained DNA sequences of mitochondrial and nuclear genes and performed phylogenetic and phylogeographic analyses. Divergence time estimation and ancestral area reconstruction were also carried out to propose a biogeographical hypothesis, and species boundaries within the species-group were also tested.

**Results:**

We identified four clades within the *Pantosteus plebeius-nebuliferus* species-group in both markers. Divergence ranged from 5.9% to 9.2% for *cytb* and 0.1% to 0.9% for *GHI*. We observed significant genetic structure and no shared haplotypes between clades. We estimated that the clades diverged during the last 5.1 Myr, with a biogeographic scenario suggesting eight vicariant and four dispersal events through the historic range of the species-group. We found that the best species-delimitation model is when four species are assumed, which correspond to the main clades. We identified nine evolutionary significance units (ESUs), pertinent to the conservation of the group, each representing populations present in distinct drainage basins.

**Conclusions:**

The evolutionary history of the *Pantosteus plebeius-nebuliferus* species-group is characterized by vicariant post-dispersal processes, linked to geological changes in the Sierra Madre Occidental and central Northern Mexico since the Pliocene. This is congruent with biogeographic patterns described for other co-distributed fish species. We propose a new phylogenetic hypothesis for the species-group, clarifying the taxonomy of this evolutionarily complex group. Our results suggest that the species-group consists of at least four clades with independent evolutionary histories, two of which may represent new undescribed species. Our identification of ESUs provides a basis upon which conservation measures can be developed for the species-group.

**Electronic supplementary material:**

The online version of this article (10.1186/s12862-018-1286-y) contains supplementary material, which is available to authorized users.

## Background

The structure of current North American biodiversity is related to geological (tectonic and volcanic) and climatic changes that occurred mainly during the Neogene (*ca.* 33 Million years ago (Mya) and Quaternary (*ca.* past 2.5 Mya) [1, 2]. The Sierra Madre Occidental (SMOC) mountain range in Mexico is an area of high faunal and floristic endemism, and is considered an important biogeographic corridor [3]. The formation of the SMOC during the Oligocene (*ca.* 33-23 Mya) until the present [2, 4] is an important factor influencing the evolutionary history of several taxa, particularly organisms with low vagility such as freshwater fish species [3, 5–12].

Although the biodiversity of northwestern Mexico is mainly related to tecto-volcanic activity of the Tertiary, the SMOC is also considered a Pleistocenic refuge, and associated with the expansion and contraction of the distribution of numerous species [13]. The effects of climate changes on epicontinental waters has significantly affected the distribution of the fish fauna of this region [3]. The evolutionary history of the sucker fish family Catostomidae in Mexico may have been influenced by these processes, especially in the case of the Catostominae subfamily, the predominant fish group of the family in Mexico [14].

**Fig. 1:**
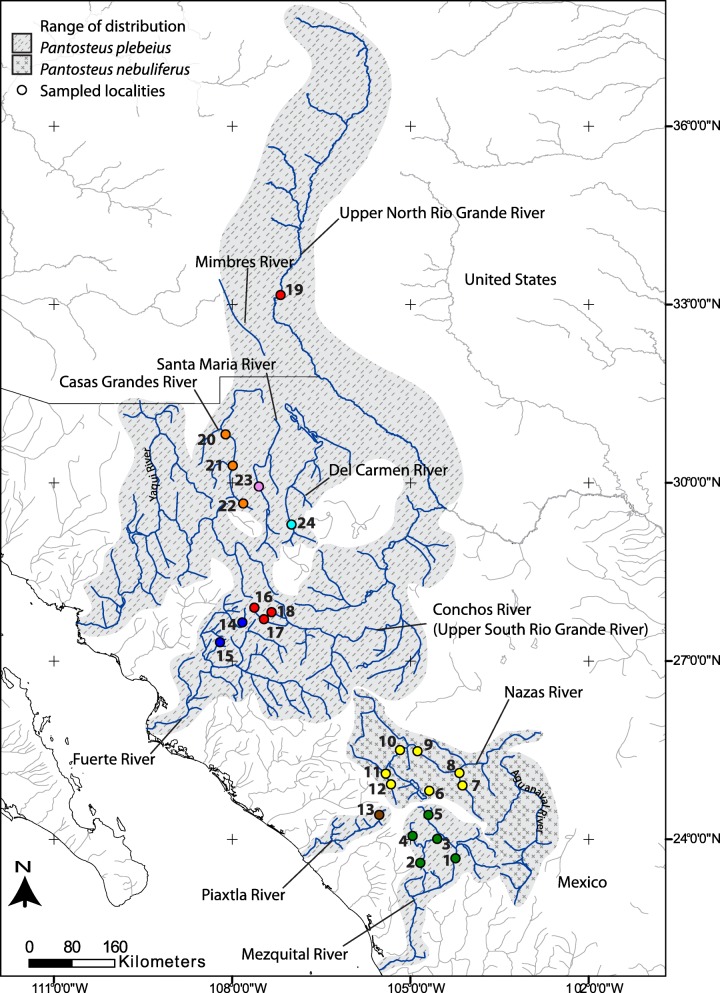
Distribution of *Pantosteus plebeius-nebuliferus* species-group and sampling localities. The colours correspond to the eight main basins and numbers to the localities: (1) Santa Gertrudis, (2) Arroyo Las Bayas, (3) Pino Suarez, (4) La Barranca, (5) Puente Mimbres, (6) El Cuarto, (7) Peñón Blanco, (8) Covadonga, (9) Arroyo Torreones, (10) El Peñasco, (11) El Olote, (12) Atotonilco, (13) Las Vegas, (14) Fuerte, (15) Oteros, (16) Bocoyna, (17) Conchos, (18) Ureyna, (19) South Fork Palomas Creek, (20) Escalariado, (21) Casas Grandes, (22) Ignacio Zaragoza, (23) Buenaventura and (24) Santa Clara

Within the Catostominae, two closely related species of the genus *Pantosteus* Cope & Yarrow, 1875; inhabit northern Mexico including the SMOC: *Pantosteus plebeius* (Baird & Girard, 1854) and *Pantosteus nebuliferus* (Garman, 1881) [14, 15]. *Pantosteus nebuliferus* has a restricted range, and is endemic to the endorheic Nazas and Aguanaval drainage basins. In contrast, *Pantosteus plebeius* is widespread and occurs in several drainage basins across the SMOC: the Mezquital, Piaxtla, Fuerte, and Yaqui basins; the Central Guzman hydrographic system (Santa Maria, Casas Grandes, and Del Carmen basins); the Upper Rio Grande basin (Conchos River in Mexico and Rio Grande River in USA); and the Mimbres basin in New Mexico, USA (Fig. 1) [16]. *Pantosteus plebeius* is also distributed in the Gila River of the Colorado basin, but the origin of this population is uncertain, some consider it to have been artificially introduced [17, 18], while others suggest this population is the result of stream capture from the Mimbres River [19].

As with other *Pantosteus* species in Mexico, *P. plebeius* is believed to have colonized several drainage basins during the postglacial period [20]. Dispersal during anastomosis of rivers, followed by vicariance after river isolation, has been hypothesized as the main factor influencing genetic divergence among *Pantosteus* populations. It has been suggested that the populations of some drainage basins could represent one or more independent evolutionary lineages, such as those of the Mezquital, Piaxtla, Fuerte, and upper Rio Grande basin, each displaying significant genetic structure compared with Mimbres and Gila River populations [21, 22]. Given the complex evolutionary history of *P. plebeius* it has been suggested that this lineage may represent several distinct species, one or more of which may be synonymous with *P. nebuliferus* [23].

*Campostoma ornatum* (Girard, 1856) and *Codoma ornata (*Girard, 1856) are codistributed with the *Pantosteus plebeius-nebuliferus*c species-group throughout its range. Phylogeographic studies of these species have shown repeated isolation scenarios followed by merge or convergence in response to range expansion (reticulated biogeographic history) [3, 11]. This pattern was related to repeated events of dispersal and isolation associated with tecto-volcanic activities occurring in the SMOC since the early Pliocene, as well as to climatic fluctuations during glacial and interglacial periods. Population structure in *Campostoma ornatum* and *Codoma ornata* is reflected in high genetic differentiations among populations inhabiting the drainage basin in the northern SMOC, including the Guzman system, and the Río Grande, Yaqui, Mayo, Fuerte, and Nazas basins [3, 11].

The aim of this study was to 1) examine the phylogeography and reconstruct the evolutionary history of the *Pantosteus plebeius-nebuliferus* species-group in Mexico; 2) to explore the genetic diversity and structure of the species-group; and 3) determine population divergence times using nuclear and mitochondrial markers. We hypothesize that the genetic structure and distribution of the main clades of *Pantosteus plebeius-nebuliferus* are associated with the geological history of the SMOC and central Northern Mexico.

## Methods

### Sampling and dna isolation

We collected 87specimens with permission of relevant authorities in July 2013 from 20 localities in 8 drainage basins (Fig. 1 and Table 1) using electrofishing and hand nets. We included 6 samples from 4 localities from GenBank (GU937833, KJ441235 and KJ441237-KJ441240) for a total of 24 localities in 9 basins (localities 14, 18, 19 and 20 in Fig. 1).

**Table 1:** Sampled populations of the *Pantosteus plebeius-nebuliferus* species-group

Species	Basin	*cytb* individuals	*GHI* individuals	GenBank Accession
*Pantosteus nebuliferus*	Nazas	29	29	*cytb*: MG203677-MG203705 *GHI* allele 1: MG203712-MG203736, MG203784-MG203787 *GHI* allele 2: MG203794-MG203820, MG203868-MG203869
*Pantosteus plebeius*	Mezquital	12	8	*cytb:* MG203619-MG203626, MG203669-MG203672 *GHI* allele 1: MG203706-MG203711, MG203770-MF203771 *GHI* allele 2: MG203788-MG203793, MG203860-MG203861
	Piaxtla	4	4	*cytb*: MG203673-MG203676 *GHI allele 1*: MG203772-MG203775 *GHI* allele 2: MG203862-MG203865
	Fuerte	11	9	*cytb*: MG203627-MG203636, KJ441240 [61] *GHI* allele 1: MG203737-MG203744, MG203783 *GHI* allele 2: MG203821-MG203829
	Uppers North R. Grande (Palomas)	1	1	*cytb*: KJ441237 [61] *GHI*: GU937833 [105]
	Upper South R. Grande (Conchos)	2	2	*cytb*: MG203637, KJ441239 [58] *GHI* allele 1: MG203745-MG203746 *GHI* allele 2: MG203830-MG203831
	Del Carmen	10	9	*cytb*: MG203658-MG203657, KJ441238 [61] *GHI* allele 1: MG203760-MG203767, MG203776 *GHI* allele 2: MG203849-MG203857
	Santa Maria	8	8	*cytb*: MG203650-MG203666, *GHI* allele 1: MG203756-MG203759, MG203777-MG203779, MG203782 *GHI* allele 2: MG203842-MG203848, MG203867
	Casas Grandes	15	13	*cytb*: MG203638-MG203649, MG203667-MG203668, KJ441235 [61] *GHI* allele 1: MG203747-MG203755, MG203768-MG203769, MG203780-MG203781 *GHI* allele 2: MG203832-MG203841, MG203858-MG203859, MG203866

Despite a considerable sampling effort, we were unable to obtain any specimens from the Aguanaval and Yaqui basins. We obtained fin clips and preserved them preserved in absolute ethanol and stored them at -75 °C. We fixed several specimens in formalin for identification and deposited in the Fish Collection of the Universidad Michoacana de San Nicolas de Hidalgo CPUM, Morelia, Mexico. We released the remaining captured fish alive.

We performed isolation of genomic DNA with BioSprint DNA Blood Kit QIAGEN according to the manufacturer’s instructions. We amplified the complete cytochrome *b* mitochondrial gene (*cytb*) using the primers GLuDG [24] and H16460 [25] and the nuclear 3^rd^ intron of the growth hormone copy I (*GHI*), using the primers GHI3F and GHI3R [26].

We amplified DNA samples by Polymerase Chain Reaction (PCR), using 12 μl volume reactions with final concentrations of 0.2 μM of each primer, 0.25 mM of each dNTP, 1.5 M of MgCL_2_, and 1 U of *Taq* DNA Polymerase. The PCR procedure consisted of 2 min at 95 °C followed by 35 cycles of 45 s at 94 °C for denaturation, 1.5 min at 46 °C and 52.2 ° C for primer alignment for *cytb* and *GHI* respectively, 2 min at 72 °C for synthesis, and a final extension of 5 min at 72 °C. We quantified the PCR products by electrophoresis on 1.5% agarose gel and submitted to Macrogen Inc. (Korea) and htSEQ Inc (High-Throughput Sequencing, University of Washington, USA) for sequencing.

### Genetic variation

We estimated the genetic diversity within drainage basins for the two gene markers based on nucleotide diversity (π), haplotype diversity (h), and the proportion of segregating sites (Θ_S_), using the software DNAsp 5.0 [27]. We obtained the genetic *p*-distances (D_P_) between populations using Mega 5.2 [28]. A bootstrapping process was implemented with 1000 repetitions.

We estimated the genetic structure at the interspecific and intraspecific level of the *Pantosteus plebeius-nebuliferus* species-group with fixation indices (Φ_ST_) for both molecular markers. We used spatial analysis of molecular variance of geographically homogeneous K groups (SAMOVA) with SAMOVA 2.0 [29] and AMOVA (for K=1 to identify genetic structure without *a priori* information and the genetic variance explained within population) with Arlequin 3.5.1.3 [30] to define groups of populations (basins) that were maximally differentiated without constraints of their geographic distribution. For both SAMOVA and AMOVA analysis we used 10 000 iterations from each of 100 random initial conditions. We tested for each K value from 2 to 8 in SAMOVA. We applied a Bonferroni correction [31] to each *p*-value obtained in the paired test of genetic differentiation.

### Phylogenetic analysis and haplotype network reconstruction

We manually aligned DNA sequences in Mega 5.2 and examined them using chromatograms. We phased *GHI* sequences with point mutations using DNAsp and applied a test of recombination using a coalescent-based Bayesian method (10 000 replicates) in the same software.

We used two algorithms for phylogenetic reconstructions, using *Catostomus catostomus* as outgroup (GenBank Accession: AF454871 for *cytb* and GU937824 for *GHI*). We conducted independent Maximum Likelihood (ML) analyses with RAxMLGUI 1.3.1 [32, 33] for both genes, performing 10 000 bootstrap repetitions and using the evolutionary substitution model estimated with PartitionFinder [34]. These models were General Time-Reversible [35] + gamma (GTR+G) for the complete *cytb* and Tamura-Nei [36] + gamma (TrN+G) for *GHI* (see Additional file 1). We conducted Bayesian phylogenetic (BI) reconstruction with the software MrBayes 3.2.6 [37], using the above selected evolutionary substitution model and implementing two runs of four Markov Chain Monte Carlo (MCMC) processes with 7 million generations for *cytb* and 15 million generations for *GHI*, sampling every 100 generations in both cases. The difference in chain length was because *GHI required* more generations to obtain sufficient values of effective sample size (> 200). We evaluated the convergence of the log-likelihood (-InL) values of the two runs, with 10% of reconstructions discarded as burn-in, to construct the consensus tree (σ < 0.005). We used the posterior probabilities obtained based on a confidence limit of 95% (highest posterior density-HPD) to evaluate the support of nodes. We used a incongruence length difference test (Partition homogeneity test) [38] in PAUP* 4.0b10 [39] to evaluate the significance of conflict among data sets. We used 1000 resampling characters, and performed concatenated analyses of nuclear and mitochondrial data for ML and BI with the parameters previously mentioned. We constructed haplotype networks for both genes using PopART (available at htt://popart.otago.ac.nz) and applying the Median-Joining method [40].

### Divergence times estimation and ancestral area reconstruction

We estimated divergence times by a coalescent-based method using the software BEAST 1.8.0 [41], based on the described evolutionary substitution model for both genes (see Additional file 1). We used a relaxed molecular clock, using *Beast (including nuclear an mitochondrial data) with Yule speciation tree model. The clock was calibrated using the fossil record (in hard minimum bound for Lognormal distribution), which was located in the root of the *Pantosteus plebeius-nebuliferus* species-group. This was represented by fossils of *Pantosteus asitus*, dated from the Miocene-Pliocene (7.5-2.5 Mya) [42]. We conducted another molecular clock analysis using the mutation rate range for *cytb* estimated for teleosts of 0.76-2.2%/Myr with uniform distribution in BEAST [43–46].

We implemented three independent *Beast analyses, each with 70 000 000 generations, sampling every 1000 generations. After evaluating the posterior parameter values based on effective sample size, and according to the convergence data, using Tracer 1.6 [47], we discarded 10% of runs and combined the three analyses to construct a maximum clade credibility tree using BEAST modules (LogCombiner and TreeAnnotator, respectively).

We performed an ancestral area reconstruction with RASP 3.2, 20160719 [48], applying Statistical-Dispersal Vicariance Analysis (S-DIVA) [49, 50] and the Statistical Dispersal-Extintion-Cladogenesis model (S-DEC) [51]. In both cases we used the chronograms obtained in BEAST to resample every 3000 samples from the total set to obtain a subset of 70 000 trees. We considered nine areas in the analysis corresponding mainly to the drainage basins sampled The areas included were the basins: A, Upper North Rio Grande (Palomas); B, Casas Grandes; C, Santa Maria; D, Del Carmen; E, Fuerte; F, Upper South Rio Grande (Conchos); G, Piaxtla; H, Nazas; and I, Mezquital. We applied an unconstrained model allowing any combination of geographic range in adjacency matrix with a maximum of five adjacent hydrographic basins, configuring the dispersal rate with the same probability for each population (basin) of the *Pantosteus plebeius-nebuliferus* species-group.

### Species delimitation analysis

We performed species delimitation tests using Bayesian Phylogenetics and Phylogeography software BPP 3.4 [52] to generate posterior probabilities for two species-delimitation models in the *Pantosteus plebeius-nebuliferus* species-group,. We used the reversible-jump Markov chain Monte Carlo (rjMCMC) methodology [53] to implement an species delimitation analysis *A10* in BPP using a fixed guide tree (in this case a species tree) for a species-delimitation model assuming four species according with the main clades observed, which was obtained in the divergence time estimation as mentioned above. We provided an additional guide tree for a species-delimitation model assuming two species, where we assigned individuals to lineages defined by recognized species *P. plebeius* and *P. nebuliferus* according with the distribution described for these species [16]. We performed an analysis *A11* for unguided species delimitation analysis to determine the best species-delimitation model compared to *a priori* models mentioned above under *A10* analyses.

Including both molecular markers, we analyze three combinations of parameters for population size (*Θ*) and species divergence time (τ): In large population size with deep divergences (*Θ α*=1, *β*=10; τ *α*=1, *β*=10); small population size with low divergences (*Θ α*=2, *β*=2000; τ *α*=2, *β*=2000); and large population size with low divergences (*Θ α*=1, *β*=10; τ *α*=2, *β*=2000). We ran the analyses for 500 000 generations, sampling each five generations, with a final burn-in of the first 25 000.

## Results

We obtained a total of 92 DNA sequences of the cytb gene (1140bp) (Genbank accession numbers: MG203619-MG203705) and 83 of the 3^rd^ intron of GHI (654bp) (Genbank accession numbers: MG203706-MG203869) for Pantosteus plebeius (*n* = 63 of cytb and *n* = 49 of GHI) and P. nebuliferus (*n* = 29 of cytb and *n* = 33 of GHI) from 22 localities in 8 drainage basins, representing most of the species distribution range [16]. We sequenced 87and 82 sequences respectively for cytb and GHI and obtained5 of cytb and 1 for GHI from GenBank (Fig. 1 and Table 1). We did not detect any significant recombination in the nuclear GHI sequences (*p* > 0.05).

### Genetic diversity

Although the haplotype diversity in all basins was relative high (h = 0.829-0.981) for *cytb* and *GHI*, the nucleotide diversity and the proportion of segregating sites were low for both markers (π = 0.001-0.010 and Θ_S_ = 0.0005-0.010) (Table 2). The populations with the lowest nucleotide diversity (π = 0.003) were those from Fuerte basin. The Casas Grandes and Nazas basin populations showed the highest number of haplotypes. However, the number of haplotypes was different in each marker by population as result of the difference in variation that each molecular marker has.

**Table 2:** Genetic diversity for *cytb*|*GHI* markers in *Pantosteus plebeius-nebuliferus* populations

Basins	Hn	π	h	Θ_S_	
Nazas	12|18	0.010|0.003	0.850|0.829	0.009|0.003	
Mezquital	8|3	0.008|0.001	0.924|0.342	0.010|0.001	
Piaxtla	1|3	--|0.008	--|0.464	--|0.001	
Fuerte	7|2	0.003|0.001	0.873|0.471	0.006|0.0005	
Upper South R. Grande (Conchos)	2|2	0.003|0.001	1|0.500	0.003|0.001	
Upper North R. Grande (Palomas)	1|1	--	--	--	
Del Carmen	5|2	0.005|0.001	0.800|0.503	0.006|0.0005	
Santa Maria	7|7	0.005|0.006	0.964|0.792	0.005|0.003	
Casas Grandes	13|6	0.006|0.002	0.981|0.683	0.008|0.002	

*Hn* number of haplotype, π nucleotide diversity, *h* haplotype diverisity, Θ_*S*_ proportion of segregating sites

### Genetic distances and structure

The highest absolute genetic distances (AGD) for *cytb* found between clades were between the Mezquital (Clade IV) and Piaxtla/Nazas basins (Clade III) (D_P_ = 9.2%) (Table 3), and higher AGD than 5% between all clades was observed. Based on *GHI* the AGD highest distances (D_P_ = 0.9%) were between the Mezquital and Fuerte and Del Carmen populations, and between the Piaxtla and Del Carmen and Fuerte populations. Between all clades AGD distances were lower than one percent. The lowest AGD were observed between populations of Casas Grandes and Santa Maria populations for *cytb* (D_P_ = 1.3%) and between the Palomas and Casas Grandes and Santa Maria for *GHI* (D_P_ = 0.1%), both comparisons within the Clade I (Guzman hydrographic system).

**Table 3:** Absolute pairwise un-corrected *p*-distances D_P_ for *cytb* (under the diagonal) and *GHI* (above the diagonal using pairwise-deletion) of populations of *Pantosteus plebeius-nebuliferus* species-group

	Mezquital	Fuerte	USR Grande (Conchos)	Casas Grandes	Santa Maria	Del Carmen	Piaxtla	Nazas	UNR Grande (Palomas)
Mezquital	--	0.9	0.8	0.8	0.8	0.9	0.7	0.8	0.6
Fuerte	8.7	--	0.1	0.7	0.7	0.8	0.9	0.7	0.5
UNR Grande (Conchos)	8.8	0.6	--	0.6	0.6	0.7	0.8	0.7	0.5
Casas Grandes	8.6	5.7	5.9	--	0.2	0.2	0.7	0.6	0.1
Santa Maria	8.1	6.6	6.6	2.6	--	0.2	0.8	0.7	0.1
Del Carmen	7.9	5.7	5.7	1.3	2.0	--	0.9	0.7	0.2
Piaxtla	8.9	7.6	7.2	7.6	7.9	7.5	--	0.4	0.6
Nazas	9.3	8.1	7.7	7.8	7.9	7.8	1.4	--	0.5
USR Grande (Palomas)	8.5	6.3	6.3	1.7	2.6	1.6	7.4	8.0	

*UNR* Upper North River, *USR* Upper South River

AMOVA revealed significant genetic structures among populations (Φ_ST_ = 0.9028 for *cytb*; Φ_ST_ = 0.6904 for *GHI*; *p* < 0.05) (Table 4). The highest genetic structure with SAMOVA was found when K = 8 (Φ_CT_ = 0.8784 for *cytb*; Φ_CT_ = 0.7460 for *GHI*, *p* < 0.05), and each basin was clustered in an independent group, with the exception of one population of the current Upper South Rio Grande, the Conchos population, which was clustered with the Fuerte population. In the case of *GHI*, no significant genetic structure was observed when groups of populations (basins) recognized for *P. plebeius* and *P. nebuliferus* were compared (Φ_CT_ = 0.1707, *p* > 0.05).

**Table 4:** Spatial Analysis of Molecular Variance (SAMOVA) of the *Pantosteus plebeius-nebuliferus* species-group baes on *cytb*|*GHI*. Only the results for K = 2, 4, and 8 are shown, as they are meaningful in phylogenetic, systematic, and biogeographic terms

Correspondence	K	Groups	Φ_CT_	Φ_SC_	Φ_ST_
One genetic pool (via AMOVA)		--	--	--	0.9028*|0.6904*
*Pantosteus plebeius*/*Pantosteus nebuliferus*	2	(Palomas/Casas Grandes/Santa Maria/Del Carmen/Conchos/Fuerte/Mezquital) (Piaxtla/Nazas)	0.3545*|0.1707	0.8742*|0.6606*	0.9188*|0.7185*
Phylogenetic inference (Clades)	4	(I) (II) (III) (IV)	0.7685*|0.6166*	0.6324*|0.2715*	0.9149*|0.7207*
Best genetic structure identified without geographic information	8	(Pamolas) (Casas Grandes) (Santa Maria) (Del Carmen) (Conchos/Fuerte) (Piaxtla) (Nazas) (Mezquital)	0.8784*|0.7460*	0.2051*|0.2156	0.9033*|0.6912*

*p < 0.05

High Φ_ST_ and significant pairwise values were observed among basins in both molecular markers, with Φ_ST_ ranging form 0.461 to 0.968 for *cytb* and form 0.050 to 0.907 for *GHI* (*p* < 0.5) (Table 5). The highest value was observed between the populations of Piaxtla and Fuerte for *cytb*, while between Piaxtla and Del Carmen populations for *GHI.* The lowest but significant Φ_ST_ was between Piaxtla and Nazas for *cytb* and between Del Carmen and Santa Maria for *GHI*. In contrast to *cytb* Φ_ST_ values, no significant Φ_ST_ between populations of Santa Maria and Casas Grandes were observed for *GHI*.

**Table 5:** Genetic differentiation using pairwise Φ_ST_ for *cytb* (under the diagonal) and *GHI* (above the diagonal) among basins

	Nazas	Piaxtla	Mezquital	Fuerte	Conchos	Casas Grandes	Del Carmen	Santa Maria	Palomas
Nazas	--	**0.532**	**0.742**	**0.722**	0.656	**0.640**	**0.723**	**0.572**	0.619
Piaxtla	**0.461**	--	**0.875**	**0.897**	0.914	**0.783**	**0.907**	**0.596**	0.900
Mezquital	**0.896**	**0.931**	--	**0.896**	0.899	**0.801**	**0.903**	**0.657**	0.890
Fuerte	**0.899**	**0.968**	**0.936**	--	0.050	**0.777**	**0.887**	**0.635**	0.868
Conchos	0.874	0.990	0.917	0.473	--	0.710	0.894	0.412	1.000
Casas Grandes	**0.889**	**0.940**	**0.923**	**0.921**	0.911	--	**0.314**	0.013	0.327
Del Carmen	**0.888**	**0.960**	**0.922**	**0.938**	0.934	**0.649**	--	**0.178**	0.789
Santa Maria	**0.884**	**0.951**	**0.914**	**0.936**	0.921	**0.795**	**0.777**	--	0.200
Palomas	0.872	1.000	0.908	0.951	0.956	0.655	0.763	0.780	--

Bold format = *p* < 0.05 after Bonferroni correction

### Phylogenetic relationships and haplotype network analysis

**Fig. 2:**
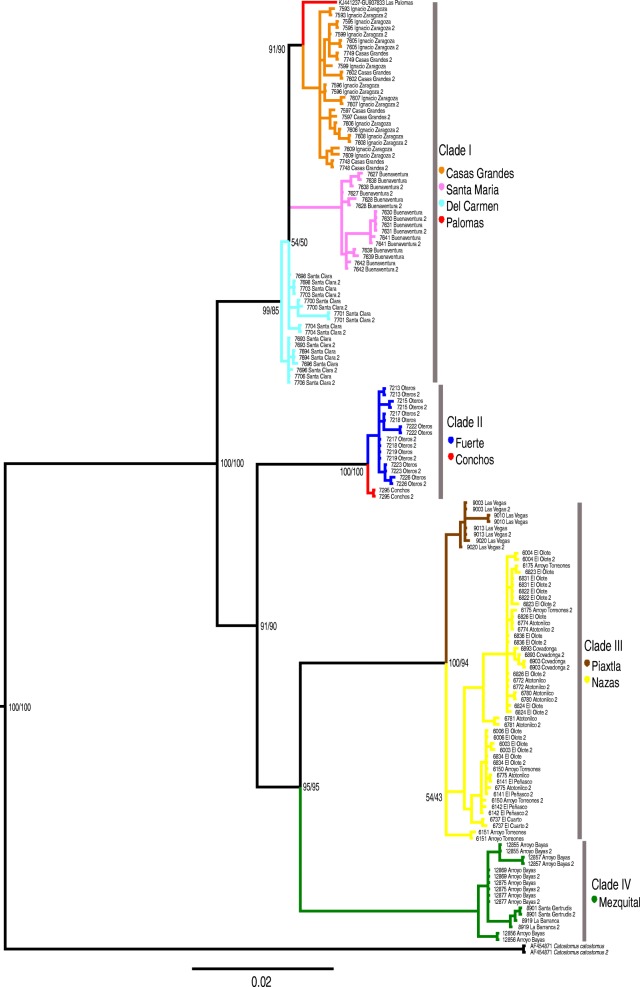
Phylogenetic reconstruction *Pantosteus plebeius-nebuliferus* species-group based on the concatenated data of *cytb* gene and *GHI* region. The numerical values in nodes represent the posterior probabilities and the bootstrap values for Bayesian Inference and Maximum Likelihood, respectively

The incongruence length difference test did not show significant differences (*p* < 0.05), indicating that both molecular markers presented the same phylogenetic signal. Bayesian and Maximum Likelihood phylogenetic analyses for the *cytb* and *GHI* sequences (see Additional files 2 and 3) and concatenated analysis (Fig. 2) recovered the same topology, with four highly supported clades (main clades). Clade I included samples from the Guzman hydrographic system (Casas Grandes, Santa Maria, and Del Carmen basins) and the Upper North Rio Grande system (Palomas). Clade II comprised the populations of the Fuerte basin and Conchos River (Upper South Rio Grande basin). Clade III, corresponding to the Nazas and Piaxtla basin populations and Clade IV consisted of the population from the Mezquital basin.

**Fig. 3:**
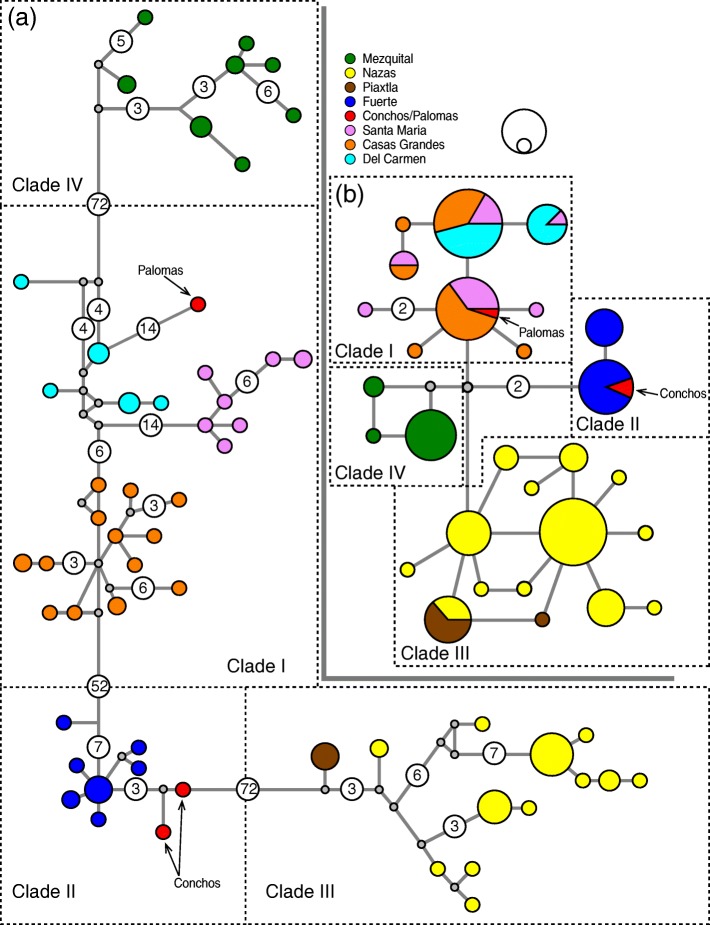
Haplotype network for *Pantosteus plebeius-nebuliferus* species-group based on *cytb* (**a**) and *GHI* (**b**). The numbers inside the circles represent the number of mutations steps between haplotypes. The grey circles represent median vector or hypothesised sequences which is required to connect existing sequences within the network

The same reciprocally monophyletic haplogroups were recovered in the haplotype networks reconstruction for both molecular markers, corresponding to the four main clades obtained by phylogenetic analysis, but differ in the relationships. In *cytb*, the Mezquital population (Clade IV) is related (72 mutations steps or MS) to the Guzman System/Palomas populations (Clade I), whereas the Nazas/Piaxtla populations (Clade III) are more closely related to the Conchos/Fuerte populations (72 MS) (Clade II) than to the Mezquital population (Fig. 3a and Fig. 3b). The number of MS separating the population of Fuerte from the Conchos River; and the population of Nazas from Piaxtla River, was 3 MS in both cases. We did not include as MS the median vectors or hypothetical haplotypes implied by the haplotype network reconstruction.

In case of *GHI*, the range of MS between clades was 1-2 MS, geographic correspondence was observed, and shared haplotypes were identified between populations that are closely related phylogenetically (Casas Grandes/Del Carmen/Santa Maria/Palomas, Fuerte/Conchos and Piaxtla/Nazas) (Fig. 3b).

### Divergence times and ancestral area reconstruction

**Fig. 4:**
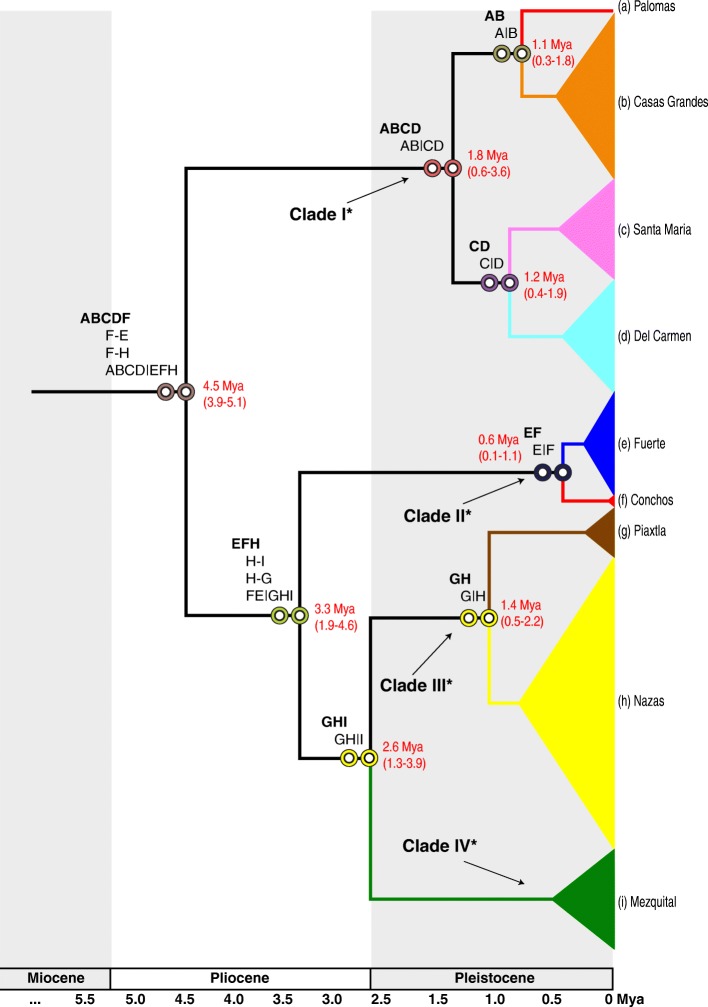
Divergence times estimation (based on species tree), S-DIVA and S-DEC analyses of the *Pantosteus plebeius-nebuliferus* species-group. Red labels represent the divergence times in millions years ago. The bold names represent the ancestral area estimated. Dispersal events and vicariant events are represented with - and | symbols, respectively. The * represent the value for posterior probability of 100% for each clade tested in the most sensitive species-delimitation model (four species) with values of *Θ α*=1, *β*=10; *τ α*=1, *β*=10 in the species delimitation analysis *A10* and *A11*

The global likelihood scores with the highest probabilities were obtained using the S-DEC approach (Fig. 4). However total congruence in ancestral areas predicted was observed between both analyses. The scenario showed by the two models (S-DIVA and S-DEC) inferred an ancestral area (ABCDF) of the most recent common ancestor (MRCA) of the *Pantosteus plebeius-nebuliferus* species-group formed by the Guzman system, and the Upper South and North Rio Grande (Conchos and Palomas, respectively). From this first vicariant event dated at *ca.* 5.1-3.9 Mya (HPD ≥ 95%) to the upper Pliocene, a succession of 7 vicariant and 4 dispersal events were identified during the historical distribution of the species (Fig. 4).

### Species limits

The species-delimitation model assuming four species was strongly sensitive to low parameter of *Θ* and τ, showing high posterior probability for the nodes in *A10* and was the species-delimitation model in *A11* analysis with the highest probability (P=1 S=4) (Fig. 4). The four species supported correspond to the main clades identified in this work for *Pantosteus plebeius-nebuliferus* (Fig. 2). The model of species-delimitation reducing the number of species to two (*P. plebeius* and *P. nebuliferus*) showed low posterior probabilities for the three combinations of parameters of *Θ* and τ (results not showed).

## Discussion

The main forces shaping cladogenetic events in the *Pantosteus plebeius-nebuliferus* species-group are consistent with geological processes occurring since the Pliocene associated with the formation and evolution of the SMOC, central North Mexico and the tectonic activity of the Rio Grande Rift. Our results revealed that the species-group comprises more than two independent evolutionary lineages with significant genetic structures between them in nuclear and mitochondrial markers, indicating a long history of isolation. Within clades mitochondrial information showed high divergence and several mutational steps between clades, unlike the nuclear marker that showed shared haplotypes between some basins; and low number of mutational steps between clades, reflecting the combined effects of low variation and incomplete lineage sorting on the nuclear loci [54]. As has occurred with the genus *Moxotoma* [12] and *Campostoma ornatum* [3], relevant vicariant events and probably river captures may have played an important role in range expansion shaping the spatial distribution of genetic variation in some drainage basins. Genetic variation within drainage basins seems to have been influenced by ecological characteristics of the species and climatic fluctuations since the Pleistocene. Further studies should seek to identify the roles of historical, intrinsic, and anthropogenic influences on genetic differentiation within basins [55].

### Historical biogeography of pantosteus plebeius-nebuliferus

#### Pliocene events

**Fig. 5:**
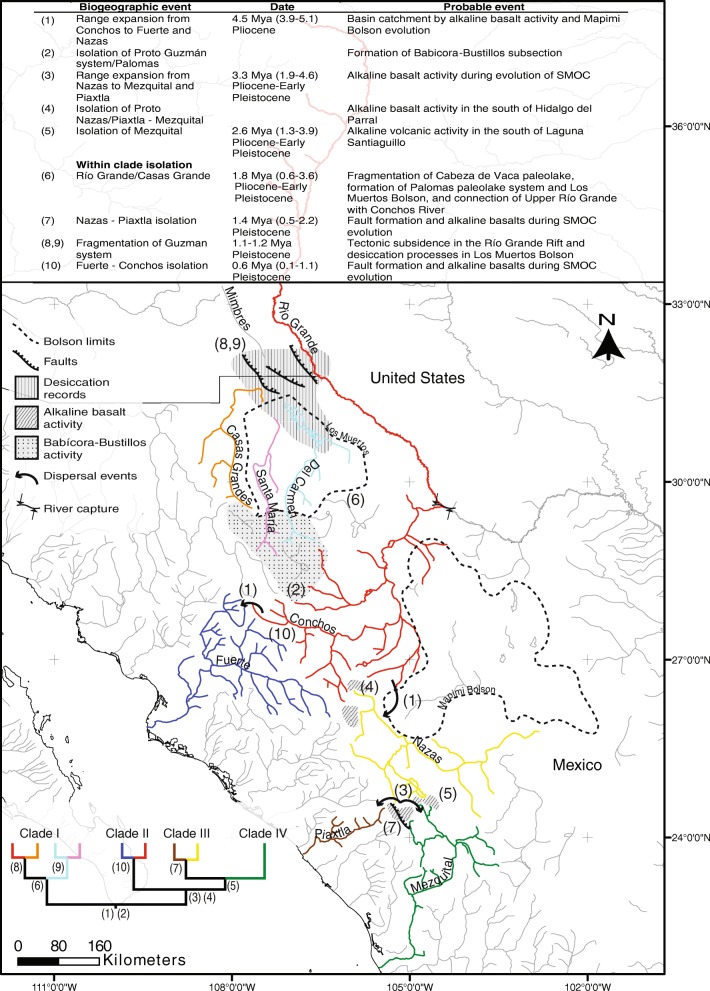
Biogeographical scenario for the *Pantosteus plebeius-nebuliferus* species-group in the North Mexico

We dated the most recent common ancestor of all clades of the *Pantosteus plebeius-nebuliferus* species-group to the Pliocene (5.1-3.9 Mya, HPD ≥ 95%) (Fig. 5), in an ancestral area comprised by the proto-Guzman system, proto-Upper South Rio Grande (Conchos) and proto-Upper North Rio Grande (Palomas). We identified a range expansion of the species-group to the Fuerte basin, which probably resulted from a basin catchment, allowing faunal interchange as proposed for *Gila pulchra* [10], and *Codoma ornata* [11]. These events occurred during the tecto-volcanic episodes in SMOC evolution, including repeated volcanic activity called “alkaline basalt events” [4, 56, 57]. Another dispersal event from the ancestral areas to Nazas was proposed. These events could have been promoted by alkaline basalts events during the Mapimi Bolson formation (Fig. 4 and Fig. 5) [12, 58, 59], which is partially congruent with patterns established for *Codoma ornata* [11] and *Campostoma ornatum* [7].

These dispersal events were followed by a vicariant event that isolated the ancestor of the Proto Guzman system/Palomas (Clade I) *ca.* 4.5 Mya, associated with the volcanism in the Babicora-Bustillos sector, disrupting the connection between the proto-Fuerte/Conchos and the proto-Guzman systems/Palomas by the periodic accumulation of sediments in the Conchos Valley [12]. This ancient isolation event is reflected in the high genetic divergence between clades I and II in *cytb* (D_P_ = 6.3-7.0%, Φ_ST_ = 0.911-0.938 *p* < 0.05; Table 3) and in the well-structured haplogroups for both markers (Fig. 3). The split between clade I with respect to the clades II, III, and IV of the *P. plebeius-nebuliferus* species-group, is geographically, genetically and in some cases temporarily consistent with other co-distributed species complexes of fishes such as *Cyprinella* spp. [60], *Campostoma ornatum* [3, 61], *Cyprinodon* spp*.* [6, 62], *Gila* spp. [10], *Codoma ornata* [11] and *Moxostoma* cf. *austrinum* from Conchos [12]. This finding also corroborates the divergence time of the Rio Grande *Pantosteus* spp. population with respect to the Nazas population in a previous work [63].

The Mapimi Bolson desertification and the alkaline basalt activity at the central part of the SMOC, was likely responsible for the disconnection that promoted the vicariant event of the ancestor of the proto-Nazas/Piaxtla (Clade III)/Mezquital basin populations (Clade IV) from the proto-Fuerte/Conchos populations in the Pliocene, an event that is consistent with results found for *Codoma ornata* results [11]. Two range expansion events of the species-group from the Nazas to Piaxtla and Mezquital basins following the vicariant event, were found in the same period, which could be the result of river catchments associated with alkaline basalt events in the SMOC [4, 12, 57], an event that is consistent with results found in other aquatic fauna, such as *Campostoma ornatum* [3] and semi-aquatic snake species of the genus *Thamnophis* [64].

Due to the physiographic complexity and the occurrence of several overlapping geologic events in the region of Nazas, Piaxtla and Mezquital, is difficult to identify specific geologic events involved in the cladogenesis of clades III and IV occurred *ca.* 2.6 Mya. The isolation of the proto-Nazas/Piaxtla from the Mezquital populations possibly had its source in the alkaline volcanic activity occurring in the region during the Pliocene, especially south of Laguna Santiaguillo in the Nazas basin, in the southern region of the SMOC [4] (Fig. 4) separating the tributary El Tunal (Mezquital Basin) from the Nazas basin occurring in the Late Miocene and Pliocene [2, 65, 66].

#### Pleistocene isolation

The origin and evolution of the three basins within the Guzman system date from the Late Cretaceous and Cenozoic to the Late Pleistocene, and its complexity could explain the species diversity and genetic divergence of fishes in the region. During the Early Pleistocene, the activity of the Rio Grande Rift and the glacial stage (Kansan, Middle Pleistocene) [67] were likely involved in the fragmentation of the Cabeza de Vaca paleolake and the formation of the Palomas pluvial paleolake, a relict of the Cabeza de Vaca that was fragmented into several endorheic rivers, lagoons, and springs. Among these, the Casas Grandes and Upper Rio Grande basins were firstly isolated from Del Carmen and Santa Maria (*ca.* 1.8 Mya) by the formation of the Los Muertos Bolson [60, 68–70] (Fig. 5). The isolation of the Upper North Rio Grande, including its flow to the Gulf of Mexico and capture of the Conchos River, was dated to the Early Pleistocene [71–74], but the tectonic subsidence in the Rio Grande Rift and desertification of Los Muertos Bolson has continued during the Middle Pleistocene and the present [75–77] allowing the fragmentation of the Guzman system. This paleohydrological pattern is reflected by our data, since we date the isolation of Palomas from Casas Grandes as *ca.* 1.1 Mya and 1.2 Mya for the isolation of Del Carmen from Santa Maria (Fig. 4). This is also consistent with previous studies that found low genetic divergences between populations of *Pantosteus plebeius* from the Mimbres River (type locality of the species) and *P. plebeius* from the Palomas River (Upper North Rio Grande) [22].

The three basins within the Guzman system were isolated and reconnected several times during the Pre-Illinoian, Illinoian, and Pre-Holocene periods [78–80]. Remixing gene pools and low genetic structures also were observed in other co-ocurring fishes along the Guzman basin, including *Gila* spp. [10, 81] and *Cyprinodon* spp. [6, 62]. However, the high interbasin genetic distances and the lack of shared haplotypes for *cytb* is evidence of the ancestral isolation that occurred in Del Carmen and Casas Grandes populations of *Campostoma ornatum* (D_P_ = 2.9%; Φ_ST_ = 0.935) [3], and the significant genetic differentiation (F_ST_ = 0.380) in *Cyprinella formosa* [60, 69, 82–84] which represents an isolation event that also occurred in the Pleistocene. In contrast, the low genetic differentiation and haplotypes shared among basins for *GHI* are evidence for the low variation and of the incomplete lineage sorting or a later genetic mixing between these basins. More studies are necessary to explain the configuration of the ichthyofauna of the Guzman system.

Finally, the vicariant events involved in the isolation of the Nazas from the Piaxtla and the Conchos from the Fuerte populations, were estimated to have occurred 1.4 Mya and 0.6 Mya in the Pleistocene (Fig. 4), respectively, and are associated with the most recent volcanic eruptions and tectonic movements in the SMOC [85]. This supports the results obtained for *Campostoma ornatum* [3], *Gila pulchra* (Conchos River & Fuerte basin) [10], and *Codoma ornata* (upper Conchos River & Fuerte basin) [11] that show low genetic differentiation as well as shared haplotypes among basins as we observed in the nuclear marker (Fig. 3).

#### Within-basin differentiation

A high number of mutational steps and genetic intrabasin distances where estimated for *cytb* in the *Pantosteus plebeius-nebuliferus* species-group*.* Surprisingly, moderate between-haplotype MS values (MS = 4-7) were found within the Mezquital, Nazas, Fuerte, Casas Grandes, and Santa Maria basin populations than between the Nazas and Piaxtla (MS = 3) and Conchos and Fuerte populations (MS = 3), reflecting considerable genetic differentiation in *cytb* (Fig. 3 and Tables 3, 4, 5). The intrapopulation genetic structure in those drainage basins could be explained in part by life history traits. Migration episodes associated with environmental fluctuations on spawning sites and food availability have been extensively described in other species of Catostomidae [86–93]. Accordingly, the historic climate fluctuations in the area [94–96] could promote similar fragmentation episodes in *P. plebeius-nebuliferus* populations, which could be responsible for the isolation of migrants in separate regions within the basins.

### Taxonomic implications and conservation considerations

The *Pantosteus plebeius-nebuliferus* species-group comprises a set of distinct genetic groups, suggesting that it is composed of at least four clades with unique and independent evolutionary histories. We found cladogenesis to be associated with the high tecto-volcanic activity and climate fluctuations from the Pliocene to the present in North Mexico. This is supported by the phylogenetic relationships, species tree, species-delimitation test including both molecular markers. Also is supported by the genetic divergences observed between clades for *cytb* (see Additional file 2), which are higher than those reported among sibling vertebrate groups for *cytb* (~2%) [97–100] and between sister species within the family [98]. In the case of *GHI*, the genetic distances estimated between species of Catostomidae are 3.3% [101] taking into account different genera of the family, and which were higher than estimated in this work (Table 3, D_P_<1%). However, although the nuclear *GHI* showed a low number of mutational steps and genetic distances (Fig. 3 and Table 3), we detected significant structure (Table 4) and recovered the four clades in the phylogenetic inference (see Additional file 3) supporting the mitochondrial results. The genetic structure and divergences in the species-group were similar to those observed in other fish taxa that partially or completely occur in basins of northwest Mexico and the southwestern USA, such as *Cyprinodon* spp. [6, 62], *Gila s*pp. [10, 81], *Cyprinella* spp. [60, 69, 84], or even among populations of other considered species complexes as *Campostoma* spp. [3, 61], and *Moxotoma* spp. [12]. Thus, we recommend an integrative taxonomic revision of the *Pantosteus plebeius-nebuliferus* to recognize the four independent evolutionary lineages as different species.

The population of the Piaxtla basin was recognized as *Pantosteus plebeius* [16] and suggested as an independent evolutionary lineage [20], our results show that this population is closely related to *P. nebuliferus*, as was also demonstrated by [63]. Low genetic divergences between populations of *P. plebeius* from the type locality Mimbres River and from the Upper North Rio Grande have been observed by other authors [22, 102]. Our results show that Paloma River is closely related with Guzman system populations, thus we suggest that Clade I must be considered populations of the recognized species *P. plebeius*. The Clade II (Fuerte basin and Conchos River populations) and the Clade IV (Mezquital River population) do not represent populations of any species recognized of the species-group studied, thus we suggest that the two clades could represent two independent evolutionary lineages that must be described and recognized as new species of the genus *Pantosteus*.

The identification of the Evolutionary Significant Units (ESU) is of particular interest especially in species considered threatened [103]. Such is the case of *Pantosteus plebeius-nebuliferus* species group, which is listed as in danger of extinction in the Norm-059-SEMARNAT-2010 [104]. We identified the group of populations whitin independent evolutionary lineages, which warrant separate management or priority for conservation. Based on the high genetic divergences; number of mutation steps; phylogenetic relationships; shared haplotypes; reproductive isolation and the subsequent absent exchangeability of populations, we suggest nine ESUs corresponding to each basin where the *P. plebeius-nebuliferus* species-group inhabit: (1) Upper North Grande River, (2) Casas Grandes, (3) Santa Maria, (4) Del Carmen, (5) Fuerte, (6) Upper South Grande River basin (Conchos River), (7) Piaxtla, (8) Nazas and (9) Mezquital. The definition of these nine ESUs should aid in establishing conservation measures of the species-group and of the evolutionary lineages of which they are configured.

## Conclusions

Our study supports at least four independent evolutionary lineages in the *Pantosteus plebeius-nebuliferus* species-group. Their biogeographic history is linked to geological events occurring since the Pliocene, associated with the formation and evolution of the SMOC, central North Mexico and the tectonic activity of the Rio Grande Rift. Other freshwater fishes already studied and occurring partially or completely in the same range of northwest Mexico and the southwestern USA have similar genetic structures and divergences. We recommend an integrative taxonomic revision of the *Pantosteus plebeius-nebuliferus* species-group and propose nine ESUs for conservation purposes which are restricted to individual basins where the species-group is distributed. As an endangered species, we hope our survey contributes to the management and preservation of these fishes.

## Additional files

Additional file 1: Evolutionary substitution model and estimated parameters for *cytb* and *GHI* by Akaike Information Criterion. (DOC 35 kb)

Additional file 2: Phylogenetic tree (Bayesian Inference/Maximum Likelihood) based on mitochondrial gene *cytb* for the *Pantosteus plebeius-nebuliferus* species-group. (EPS 1046 kb)

Additional file 3: Phylogenetic tree (Bayesian Inference/Maximum Likelihood) based on nuclear gene *GHI* for the *Pantosteus plebeius-nebuliferus* species-group. (EPS 1151 kb)

## Abbreviations

AGD: Absolute Genetic Distances
AMOVA: Analysis of Molecular Variance
BI: Bayesian Inference
*ca*: Approximately
CPUM: Fish Collection of the Universidad Michoacana de San Nicolás de Hidalgo
*cytb*: Cytochrome b
D_P_: Genetic *p*-distances
ESU: Evolutionary Significance Unit
GHI: 3rd Intron of Growth Hormone copy I
GTR+G: General Time-Reversible + Gamma
h: Haplotype diversity
HN: Number of haplotype
HPD: Highest Posterior Density
-lnL: Log-likelihood
MCMC: Markov Chain Monte Carlo
ML: Maximum Likelihood
MRCA: Most Common Ancestor
MS: Mutation Steps
Mya: Million years ago
Myr: Million years
PCR: Polymerase Chain Reaction
SAMOVA: Spatial Analysis of Molecular Variance
S-DEC: Statistical Dispersal-Extintion-Cladogenesis
S-DIVA: Statistical-Dispersal Vicariance Analysis
SMOC: Sierra Madre Occidental
TrN+G: Tamura-Nei + Gamma
UNR: Upper North River
USA: United States of America
USR: Upper South River
*Θ*: Population size
Θ_S_: Proportion of segregating sites
π: Nucleotide diversity
τ: Species divergence time
Φ_ST_: Pairwise fixation indices
