# Correction to: Historical biogeography reveals new independent evolutionary lineages in the Pantosteus plebeius-nebuliferus speciesgroup (Actinopterygii: Catostomidae)

**DOI:** 10.1186/s12862-018-1321-z

**Published:** 2018-12-27

**Authors:** Diushi Keri Corona-Santiago, Omar Domínguez-Domínguez, Llanet Tovar-Mora, José Ramón Pardos-Blas, Yvonne Herrerías-Diego, Rodolfo Pérez-Rodríguez, Ignacio Doadrio

**Affiliations:** 1 Departamento de Biodiversidad y Biología Evolutiva, Museo Nacional de Ciencias Naturales, CSIC, c/José Gutiérrez Abascal, 2, 28006 Madrid, Spain; 2 Laboratorio de Biología Acuática, Facultad de Biología, Universidad Michoacana de San Nicolás de Hidalgo, Morelia, Michoacán Mexico; 3 Facultad de Biología, Universidad Michoacana de San Nicolás de Hidalgo, Morelia, Michoacán Mexico

**Correction to: BMC Evolutionary Biology (2018) 18:173**

**https://doi.org/10.1186/s12862-018-1286-y**

Following publication of the original article [1], the authors notified us that that one of the authors’ names was incorrectly spelled. In this Correction the incorrect and correct author name are shown. The original publication of this article has been corrected.

**Originally the author name has been published as:**

- José Ramón Pardons-Blas

**The correct author name is:**

- José Ramón Pardos-Blas
