# Supplementary material for: Historical biogeography reveals new independent evolutionary lineages in the Pantosteus plebeius-nebuliferus species-group (Actinopterygii: Catostomidae)
Source: BMC Evol Biol. 2018 Nov 20;18:173. doi: 10.1186/s12862-018-1286-y (PMC6245702; doi:10.1186/s12862-018-1286-y)
Supplement: Supplementary file 1 — Evolutionary substitution model and estimated parameters for cytb and GHI by Akaike Information Criterion. (DOC 35 kb) [file 12862_2018_1286_MOESM1_ESM.doc]

**Additional file 1.** Evolutionary substitution model and estimated parameters for *cytb* and *GHI* by Akaike Information Criterion.

| Gene | Evolutionary model | lnL | Gamma |
| --- | --- | --- | --- |
| *cytb* | GTR+G | -638.643 | 0.17 |
| *GHI* | TrN+G | -1453.496 | 0.33 |

Note: lnL = log likelihood
